# Design-Based Stereology of the Lung in the Hyperoxic Preterm Rabbit Model of Bronchopulmonary Dysplasia

**DOI:** 10.1155/2021/4293279

**Published:** 2021-10-06

**Authors:** Christian Mühlfeld, Henri Schulte, Johanna Christine Jansing, Costanza Casiraghi, Francesca Ricci, Chiara Catozzi, Matthias Ochs, Fabrizio Salomone, Christina Brandenberger

**Affiliations:** ^1^Institute of Functional and Applied Anatomy, Hannover Medical School, 30625 Hannover, Germany; ^2^Biomedical Research in Endstage and Obstructive Lung Research (BREATH), Member of the German Center for Lung Research (DZL), Hannover, Germany; ^3^Corporate R&D Preclinical Department, Chiesi Farmaceutici S.p.A, Via Palermo 26/a, 43122 Parma, Italy; ^4^Institute of Functional Anatomy, Charité-Universitätsmedizin Berlin, Philippstr. 11, 10115 Berlin, Germany; ^5^German Center for Lung Research (DZL), Berlin, Germany

## Abstract

Bronchopulmonary dysplasia (BPD) is a complex condition frequently occurring in preterm newborns, and different animal models are currently used to mimic the pathophysiology of BPD. The comparability of animal models depends on the availability of quantitative data obtained by minimally biased methods. Therefore, the aim of this study was to provide the first design-based stereological analysis of the lungs in the hyperoxia-based model of BPD in the preterm rabbit. Rabbit pups were obtained on gestation day 28 (three days before term) by cesarean section and exposed to normoxic (21% O_2_, *n* = 8) or hyperoxic (95% O_2_, *n* = 8) conditions. After seven days of exposure, lung function testing was performed, and lungs were taken for stereological analysis. In addition, the ratio between pulmonary arterial acceleration and ejection time (PAAT/PAET) was measured. Inspiratory capacity and static compliance were reduced whereas tissue elastance and resistance were increased in hyperoxic animals compared with normoxic controls. Hyperoxic animals showed signs of pulmonary hypertension indicated by the decreased PAAT/PAET ratio. In hyperoxic animals, the number of alveoli and the alveolar surface area were reduced by one-third or by approximately 50% of control values, respectively. However, neither the mean linear intercept length nor the mean alveolar volume was significantly different between both groups. Hyperoxic pups had thickened alveolar septa and intra-alveolar accumulation of edema fluid and inflammatory cells. Nonparenchymal blood vessels had thickened walls, enlarged perivascular space, and smaller lumen in hyperoxic rabbits in comparison with normoxic ones. In conclusion, the findings are in line with the pathological features of human BPD. The stereological data may serve as a reference to compare this model with BPD models in other species or future therapeutic interventions.

## 1. Introduction

Bronchopulmonary dysplasia (BPD) is a chronic human lung disease associated with preterm birth. In the early days, BPD was described to be the result of a combination of respiratory distress syndrome and the effects of therapeutic interventions, including ventilation and oxygen supply [1]. With the possibility of treating preterm babies with exogenous surfactant, this form of BPD (“old BPD”) has decreased; however, the progress in neonatal care has helped to keep alive very immature neonates, born between gestation weeks 24 and 28 [2]. At this stage, lung development is incomplete and needs to progress to gain full functional capacity, while at the same time, the immature lung is required to work as a gas exchanger. The conditions for further lung development are aggravated by perinatal inflammation/infection, mechanical ventilation, and oxygen supply [3]. As a result, lung development gets disrupted which is characterized by a decrease in alveolarization and (micro-)vascular maturation as well as interstitial changes, including deposition of fibrotic tissue [4, 5]. In order to distinguish this form from the previously known condition, it has been termed “new BPD.” The pathophysiology of the new BPD, in particular, the mechanisms by which alveolarization is halted, is still not fully understood. In addition, due to the chronic nature of BPD, children with this disease are affected lifelong by the consequences of disrupted lung development, and therapeutic approaches are needed to promote extrauterine lung development in extremely preterm newborns [2, 6–8].

For these purposes, preclinical experiments addressing the pathophysiology of arrested lung development and the effects of new therapeutic interventions are essential [9, 10]. Currently, several animal models are used in BPD research to mimic parts of the pathophysiological sequelae of human preterm birth. The most frequently used is the term mouse [11] or rat [12] model of BPD in which developmental arrest is achieved by exposure of the pups to a hyperoxic environment. At birth, the lungs of mice and rats are physiologically in the saccular stage of lung development which corresponds to the 24^th^ to 30^th^ gestational week in humans [11, 13]. Hyperoxia disrupts the alveolarization process which takes place after birth in rodents [14]. However, lung biology between humans and mice differs greatly, and the translation of therapeutics tested in rodent models into the clinical situation has led to disappointing results [9]. In contrast, preterm models of BPD are used in chronically ventilated lambs and baboons [15]. These models are valuable as they are much closer to the situation of human preterm newborns; however, they are not only complex, technically demanding, and expensive but also ethically challenging. In particular, the use of the baboon model is restricted to questions that cannot be addressed otherwise, e.g., last testing of a therapeutic intervention in an animal model before entering a clinical trial [16]. As a bridge between the hyperoxia-based and preterm animal models, the rabbit model of preterm birth and subsequent exposure to hyperoxic conditions has been introduced. In this model, the pups are born three days before term by a cesarean section and then exposed to 85%-95% ambient oxygen conditions [17, 18].

Morphometric methods are extremely valuable to characterize the lungs of different animal models in quantitative terms [19, 20]. At best, the resulting data can be used for statistical testing of robust parameters allowing comparisons among different animal models and therapeutic interventions. The gold standard of lung morphometry is design-based stereology, a set of methods that is aimed at obtaining, in theory, unbiased and, in practice, minimally biased quantitative data about the volume, surface area, length, and number of certain structures [21]. While useful stereological estimations have been extensively performed in the mouse model of BPD [22–24], a formal stereological characterization of the rabbit preterm/hyperoxia model is missing so far. Although morphometric analyses in the rabbit model have been carried out [25, 26], comparison of the pathophysiological events taking place in different models requires the use of a comparable morphometric methodology, i.e., design-based stereology. Therefore, the present study used a formal stereological approach according to current American Thoracic Society/European Respiratory Society standards [21] to analyse pulmonary changes in the rabbit model of BPD and to provide reference data for future studies using this animal model. The stereological data were further complemented by lung function testing to allow structure-function analysis.

## 2. Material and Methods

### 2.1. BPD Rabbit Model

Time-mated New Zealand white rabbits were obtained from Charles River Laboratories (France). All experimental procedures were approved by the local animal ethics committee and complied with the standard European regulations on animal research (no. 744/2017). For cesarean section at the 28^th^ day of gestation (early saccular stage of lung development, term = 31^st^ days of gestation), the does were sedated with intramuscular (i.m.) medetomidine 0.25 mg/kg (Domitor®, Orion Pharma, Finland). Ten minutes later, they received i.m. 20 mg/kg of ketamine (Nimatek®, Dechra, Italy) and 5 mg/kg of xylazine (Rompun®, Bayer, Germany). Subsequently, the does were euthanized with an overdose of pentothal sodium (100 mg/kg, MSD Animal Health, USA). After death occurred, the abdomen was immediately opened, and the uterus exposed to extract all pups through hysterectomy. At delivery, pups were dried, stimulated, and divided into two groups similarly as described by Salaets et al. [27]: the normoxia group where pups were housed in 21% oxygen for 7 days and the hyperoxia group where pups were housed in 95% oxygen for 7 days. All pups were placed in custom-made incubators (Okolab, Italy) at 32°C and 50-60% relative humidity. Oxygen level, humidity, and temperature were continuously monitored for the whole experiment. Pups were fed twice daily via an orogastric tube with increasing quantities of a milk replacer (Day One®, Protein 30%, Fat 50%; Fox Valley, USA), supplemented with vitamins and probiotics (Bio-Lapis®; Probiotics International Ltd, UK), whereas immunoglobulins were added only during the first 2 days of life (Col-o-Cat®, SanoBest, Netherlands). Furthermore, intramuscular vitamin K was injected on postnatal day 2 (0.002 mg/kg, Konakion Paediatric; Roche).

### 2.2. Pulmonary Artery Microultrasound Doppler Analysis

After 7 days of exposure with normoxic or hyperoxic air, the pups were anesthetized with isoflurane (2.5% in pure oxygen) and were placed in the supine position on the Vevo Imaging station (VisualSonics, Canada). Two-dimensional images of the pulmonary artery were obtained from a parasternal short axis view at the level of the aortic valve using the MX-550D transducer (40 MHz, broadband frequency 22 MHz-55 MHz, Vevo 3100). Measurements were performed offline (Vevo LAB software package V3.0). The following variables were measured: pulmonary artery acceleration time (PAT), ejection time (PET), and PAAT/PAET ratio. An average of three cardiac cycles was used for the analyses.

### 2.3. Necropsy and Lung Function Measurements

At the end of the experimental period, pups were anesthetized with ketamine (35 mg/kg) and xylazine (6 mg/kg) i.p., and lung function testing was performed using a forced oscillation technique with the flexiVent system (flexiVent; SCIREQ, Montreal, Canada) [28]. The analysis was done with the flexiVent module 2 that is suited for rodents and other small animals weighing not more than 80-85 g and that has been used previously in the preterm rabbit model by other research groups [25, 27, 28]. The following parameters were assessed: airway resistance (Rn), tissue damping (resistance, G), and tissue elasticity (elastance, H) using Primewave-8 forced oscillation and the inspiratory capacity (IC) and static compliance (Cst) using the pressure-volume perturbation. At the end of the lung function measurements, pups were euthanized with an overdose of pentothal sodium (100 mg/kg, MSD Animal Health, USA) i.p., and whole lung samples were harvested for stereological analysis.

### 2.4. Lung Tissue Preparation

Lungs of preterm born rabbits were fixed by instillation at a pressure of 25 cmH_2_O with a fixative containing 1.5% glutaraldehyde and 1.5% paraformaldehyde in 0.15 M HEPES buffer. Our inflation fixation protocol was tested and optimized in advance for inflation fixation in preterm rabbits. After at least 24 h submersion in fixative, lungs were further processed for stereological analysis. Prior to embedding in Technovit resin, lung volume measurements were assessed by volume displacement (based on Archimedes' principle), and lung tissue was sampled by applying systematic uniform random sampling [29]. This was done by cutting the lung tissue into 2 mm thick slices, and every other slice, with a random start, was embedded in Technovit resin as described previously [29, 30]. Resin-embedded lung tissue was cut either into 1.5 *μ*m thick sections and stained with toluidine blue or into two sequential sections with a distance of 4.5 *μ*m thickness and stained with orcein for disector analysis and alveolar counting [31]. Histological slides with tissue sections were then scanned and digitalized with a microscopic slide scanner (AxioScan.Z1, Zeiss, Germany) at a 20x objective lens magnification.

### 2.5. Stereological Analysis

Stereological analysis was done with the newCAST™ software (Visiopharm®, Denmark) on digitalized slides. For the estimation of parenchymal, nonparenchymal, and ductal airspace volumes, automated systematic uniform random subsampling (SURS) was performed at a 5x objective lens magnification and with a sampling fraction of 5-7% to collect the images for further analysis. A point grid consisting of 36 points with a 4-fold subsampling fraction was superimposed over the collected images and used to estimate the respective volume densities as described previously in detail [30]. Respective total volumes were assessed by multiplying the densities with the lung volume.

For the estimation of alveolar and ductal airspace, septal volume, septal thickness, and septal surface area, an automated SURS was performed at a 20x objective lens magnification and with a sampling fraction of 0.5-1%. A test line grid with 12 lines and a length per point of 9.37 *μ*m was superimposed over the images, and points and intersections were counted. Respective volume and surface densities were estimated as described previously [30], and total volume and surface areas were obtained by multiplying the densities with the parenchymal lung volume. The presence of alveolar edema was also assessed as an additional parameter within the alveolar airspace.

For the estimation of the alveolar number, the consecutive tissue sections were aligned in the Visiopharm software. An automated SURS was performed at a 20x objective lens magnification with a sampling fraction of 0.9-1.8%, resulting in approximately 100 to 140 image pairs per lung. A counting frame with an area of 35,570.40 *μ*m^2^ was superimposed over the disector image pairs, and the number of alveoli was estimated as described previously [31, 32]. Mean linear intercept lengths (Lm) were calculated by dividing four times the total parenchymal airspace volume by the septal surface area as described previously [33].

The vascular volume, vascular wall thickness, and perivascular area were estimated using a sampling fraction of 4-9% with approximately 170-200 images at a 10x objective lens magnification. A length grid with 12 × 9 lines and a length per point of 18.74 *μ*m was used to calculate the vascular volume and surface area in vessels with a diameter of more than 25 *μ*m. A point grid with 16 × 16 points was used to estimate the mean volume and thickness of the perivascular area and vascular wall in vessels with diameter of more than 25 *μ*m. Calculations were done as described previously [34].

### 2.6. Statistical Analysis

A number of 9-10 rabbits per experimental group were included in the study. Lungs that did not properly inflate during organ harvesting due to technical issues were excluded from morphometric analysis, resulting in stereological analysis of 8 lungs per experimental group. Statistical comparison between the two groups was done with Student's *t*-test using the SigmaPlot® software (SYSTAT® Software Inc.). If normality testing failed, the Mann–Whitney rank sum test was used. Results were considered significant if *p* ≤ 0.05.

## 3. Results

### 3.1. Lung Function Measurements and Pulmonary Artery Analysis with Microultrasound

**Figure 1:**
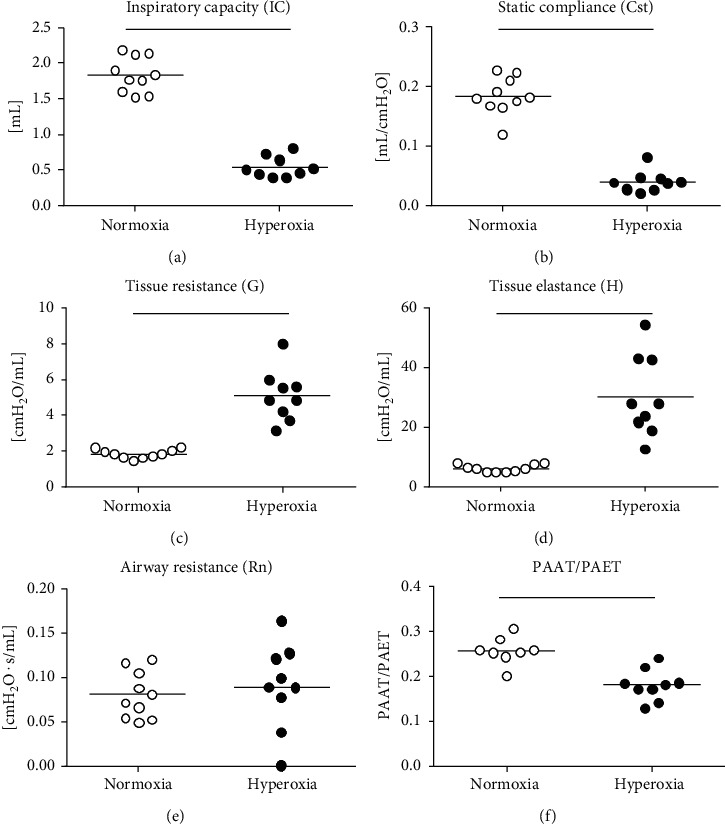
Effects of hyperoxia on lung function measurement, pulmonary arterial acceleration, and ejection time. Lung function measurements were assessed with a rodent flexiVent, measuring inspiratory capacity (a), static compliance (b), tissue resistance (c), tissue elastance (d), and airway resistance (Rn). Pulmonary artery acceleration per ejection time ratio (PAAT/PAET) was assessed with Doppler microultrasound analysis. Lines indicate statistical differences between groups (*p* ≤ 0.05), assessed with *t*-test or Mann–Whitney rank sum test if normality distribution failed.

The pups were weighed, and pulmonary artery microultrasound Doppler analysis and lung function measurements were performed prior to sacrifice. The average body weight did not differ between the two experimental groups after 7 days of exposure to hyperoxic or normoxic air and was 50 g (±4.9 g) for the normoxia-exposed pups and 49 g (±9 g) for the hyperoxia-exposed pups. However, significant differences were found in inspiratory capacity (IC), static compliance (Cst), tissue resistance (G), and tissue elastance (H) between the two experimental groups (Figures 1(a)–1(d)). While IC and Cst were significantly reduced in hyperoxia- compared with normoxia-exposed pups, tissue elastance and resistance were significantly elevated in the hyperoxic group. No differences between the groups were detected in airway resistance (Rn, Figure 1(e)), suggesting that hyperoxia treatment affected the pulmonary parenchyma but not the airways. Microultrasound Doppler analysis of the pulmonary artery furthermore revealed a decreased ratio of pulmonary artery acceleration time to pulmonary artery ejection time (PAAT/PAET) with hyperoxia treatment, suggesting development of pulmonary hypertension (Figure 1(f)). The data hence provides evidence of impaired lung function and pulmonary hypertension due to hyperoxia treatment without having an impact on body weight within the experimental duration.

### 3.2. Structural Changes in the Lung Parenchyma

**Figure 2:**
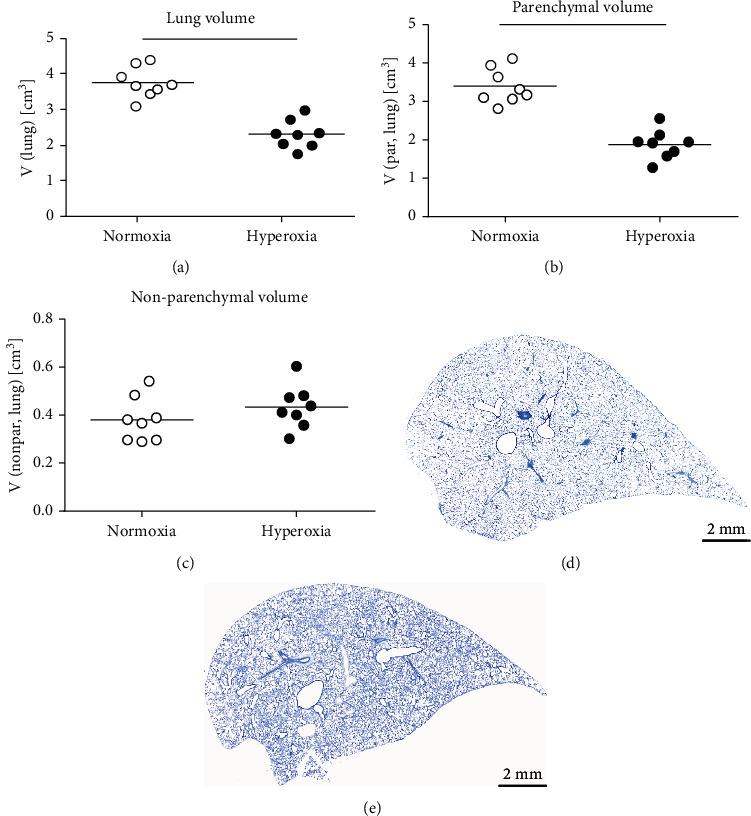
Effect of hyperoxia treatment on lung volume and parenchymal volume. Total lung volume (a) was assessed with volume displacement. Changes in the parenchymal (b) and nonparenchymal (c) lung compartments were further quantified using stereology. Representative microimages of lung samples embedded in glycol methacrylate and stained with toluidine blue are shown in images (d) (normoxia) and (e) (hyperoxia). Lines indicate statistical differences between groups (*p* ≤ 0.05), assessed with *t*-test or Mann–Whitney rank sum test if normality distribution failed.

**Figure 3:**
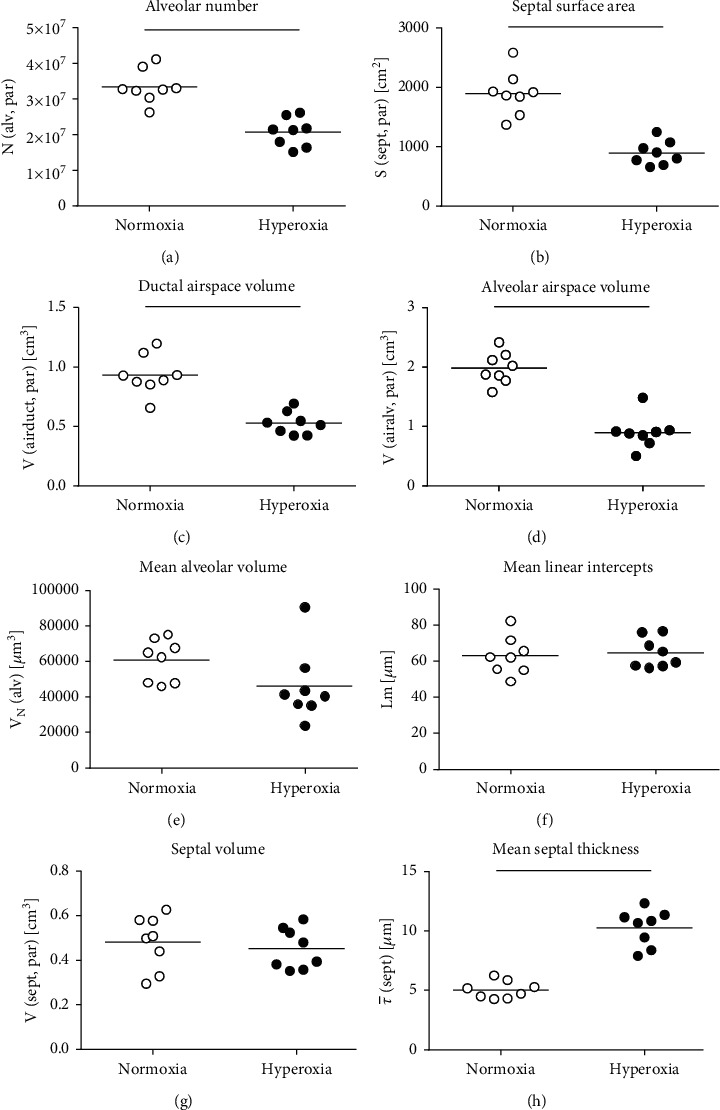
Stereological data of alveolar and septal characteristics in the lung parenchyma. Stereology was used to quantify alveolar number (a), septal surface area (b), ductal airspace (c) and alveolar airspace (d), and mean alveolar number (e) in normoxic and hyperoxic rabbit lungs. The mean linear intercept length (f) was calculated for better comparison with previous studies. Stereological quantification of septal volume (g) and mean septal thickness (h) was also performed. Lines indicate statistical differences between groups (*p* ≤ 0.05), assessed with *t*-test or Mann–Whitney rank sum test if normality distribution failed.

**Figure 4:**
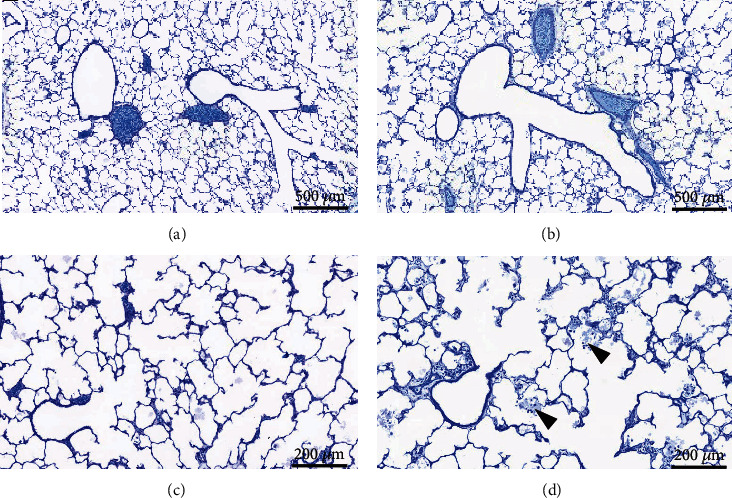
Histopathology of alveolar and septal structures in normoxia- and hyperoxia-treated rabbit lungs. Representative microscopic images of lung sections embedded in glycol methacrylate and stained with toluidine blue of normoxia- (a, c) and hyperoxia- (b, d) exposed rabbit pups. While the mean alveolar volume is similar between the two exposure groups, an increase in septal thickening and alveolar edema and inflammation (arrowheads) (d) is apparent in the hyperoxia-exposed rabbit lungs.

The lung volume, assessed with volume displacement after instillation fixation, was also significantly reduced with exposure to hyperoxia (Figure 2(a)) — similarly as the IC and the Cst. This was primarily due to changes in the parenchymal compartment of the lung that declined with hyperoxia treatment (Figure 2(b)). The total volume of the nonparenchymal lung tissue (conducting airways, vasculature, and peribronchiolar and perivascular tissues), however, was not affected by hyperoxia exposure (Figure 2(c)). Representative images providing an overview of the alterations in pulmonary structure and histopathology are shown in Figures 2(d) (normoxia) and 2(e) (hyperoxia). Stereological analysis of the parenchyma further revealed that the alveolar number as well as the septal surface area significantly declined in hyperoxia-treated rabbits (Figures 3(a) and 3(b)). Similarly, the ductal and alveolar airspace volumes were significantly reduced with hyperoxia treatment (Figures 3(c) and 3(d)). However, there was no significant difference in the mean alveolar volume and mean linear intercept lengths (Lm) between the two treatment groups (Figures 3(e) and 3(f)). The total septal volume (Figure 3(g)) did not decline with hyperoxia exposure, which, however, was mostly due to an increased septal thickening in the hyperoxia-treated lungs (Figure 3(h)).  Figure 4 shows the parenchymal lung structure in the normoxia- (Figures 4(a) and 4(c)) and hyperoxia- (Figures 4(b) and 4(d)) exposed pups at higher magnification and provides evidence of thickened alveolar septa and the presence of intra-alveolar inflammation with inflammatory cells after hyperoxia treatment.

### 3.3. Structural Changes in the Vasculature and Perivascular Area

**Figure 5:**
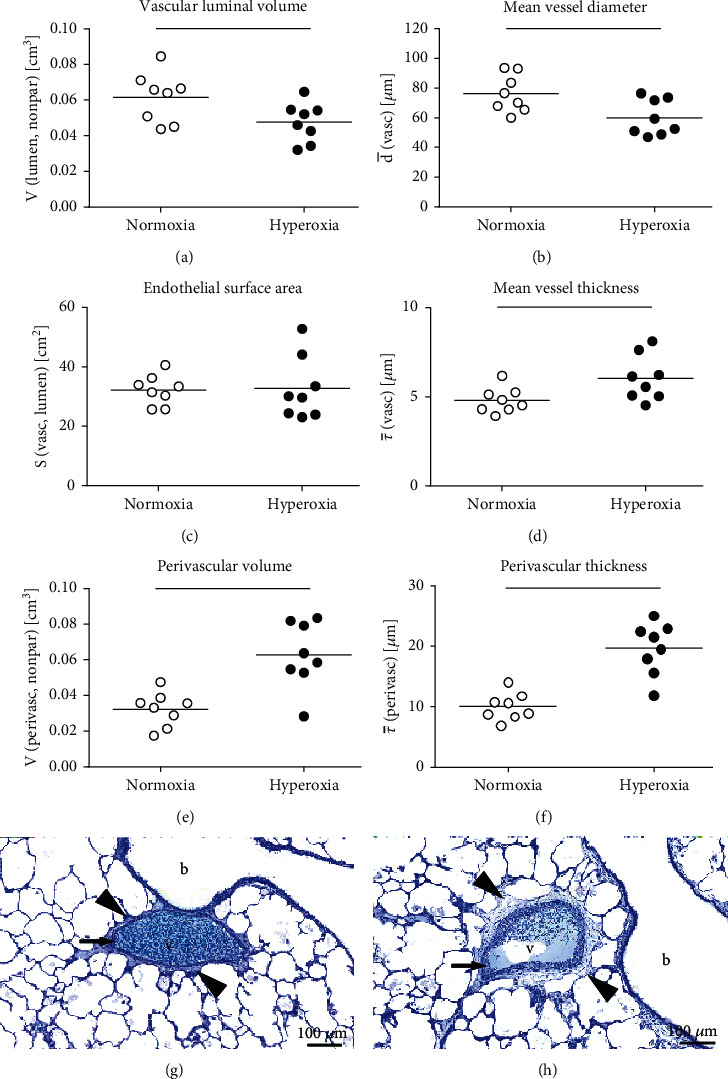
Alterations in vascular and perivascular structures. Vascular luminal volume (a), mean diameter (b), and surface area (c) as well as the mean thickness of the vascular wall (d) and the perivascular area (e) and thickness (f) were estimated using stereology. All data were collected from vessels with a diameter larger than 25 *μ*m. Lines indicate statistical differences between groups (*p* ≤ 0.05), assessed with *t*-test or Mann–Whitney rank sum test if normality distribution failed. Representative images of vessels (here, pulmonary arteries) of normoxic (g) and hyperoxic (h) lungs show increased thickening of vascular wall (arrow) and perivascular area (arrowheads). b: bronchiole; v: vessel.

Structural changes in the vasculature and the perivascular area included a decline in the vascular luminal volume (Figure 5(a)) and the mean vascular diameter (Figure 5(b)) in hyperoxia-treated lungs. The endothelial surface area of vessels with a diameter larger than 25 *μ*m (Figure 5(c)) was not affected by hyperoxia exposure. However, the mean thickness of the vessel wall, the perivascular area, and the perivascular volume (Figures 5(d)–5(f)) significantly increased with hyperoxia exposure in preterm born rabbits. The apparent differences in perivascular and vascular wall thickness are shown in representative images of pulmonary arteries of comparable size of normoxia- (Figure 5(g)) and hyperoxia- (Figure 5(h)) treated rabbits.

A summary of all acquired stereological data, including estimation of densities and total values, is provided in Table 1.

## 4. Discussion

The present study provides stereological reference data on an important animal model of BPD. In comparison with the normoxic controls, the lungs of preterm rabbit pups exposed to 95% hyperoxia were characterized by reduced alveolarization (smaller total number and surface area of alveoli, smaller total volume of parenchyma) and septal edema and/or fibroproliferation (thicker alveolar septa). In addition, the luminal volume of nonparenchymal blood vessels was reduced whereas the volume of their walls and perivascular connective tissue space was enlarged. Functionally, the lungs showed significant signs of parenchymal damage (increase in tissue resistance and elastance) in the absence of strong effects on larger airways (similar airway resistance). However, signs of pulmonary hypertension were present (decreased PAAT/PAET ratio).

Design-based stereology is the current gold standard of lung morphometry [21] because it does not depend on assumptions and provides robust data that allow for statistical testing. Design-based stereological methods have been extensively used in mouse models of BPD [22–24, 35–37] but not in the preterm rabbit model so far.

Most of the data reported in the current study are in accordance with previous morphometric studies in this animal model [25, 26] which reported similar functional data and morphometric parameters suggesting disrupted alveolarization. Although the previous studies were in line with the pathological concepts of BPD and allow comparisons between experimental groups of the same study, they are not suited to allow cross-study and cross-species comparisons. For example, Jiménez and colleagues reported a significantly higher mean linear intercept length (Lm) in the hyperoxic pups, a parameter that is mostly used as a measure of alveolar size [25]. In the present study, neither the Lm nor the mean volume of the alveoli was altered by hyperoxia. All measurements of alveolar size (Lm or mean alveolar volume) critically depend on the elastic properties of the lungs in relation to the applied fixation pressures [33]; thus, they strongly depend on the experimental setting. In addition, they are not suited to allow cross-species comparisons because of different alveolar size and elastic properties in differently sized animals. As the elastic properties may also differ among experimental groups, it is also possible that alveolar size measures can differ in the absence of alveolar number of surface area alterations.

Alveolar surface area is an important parameter to evaluate the functionally relevant diffusion area for gas exchange [38]. Its estimation, however, depends on the applied resolution and magnification, illustrated by the “Coast of Britain” phenomenon [39]. For example, surface area estimations of human lung alveoli range between 50-70 m^2^ and 130-140 m^2^ depending on whether the estimation based on light microscopy/microcomputed X-ray tomography [40, 41] or electron microscopy [38]. Tissue deformation/shrinkage during embedding can also significantly and differentially (between experimental groups) affect the results of surface area estimations [42, 43].

The most straightforward analysis of alveolarization is to estimate the total alveolar number. While originally based on geometric assumptions [44], an efficient and unbiased way of estimating alveolar number based on the disector principle [45] and the Euler-Poincaré characteristic [46] was established several years ago [32, 47]. The advantage over other morphometric methods is the possibility to compare BPD models or the influence of an experimental intervention in a certain model between laboratories and across animal species. In a rat hyperoxia model of BPD, two weeks of exposure to 60% O_2_ resulted in a significant decrease in alveolar number whereas both total alveolar surface area and mean linear intercept length were not significantly different from those of the control group [48]. In a systematic analysis of various levels of hyperoxia (21%, 40%, 60%, and 85%) or differing periods of exposure to 85% oxygen, Nardiello et al. [14] provided stereological data for the mouse model of BPD. After exposure to 85% hyperoxia from postnatal days P4 to P7, the number of alveoli was reduced to 68% of controls, whereas alveolar surface area and Lm were not significantly different. Longer hyperoxia exposure (from P1 to P7), however, decreased alveolar number to 43% of controls which was accompanied by decreased alveolar surface area and increased Lm.

In the present study, the alveolar number was reduced to 68% of control lungs with no changes in Lm or mean alveolar volume, which corresponds well with the data for P4 to P7 hyperoxia exposure in the mouse study by Nardiello et al. [14]. In contrast to their study, however, the total surface area was significantly smaller in our hyperoxia group than in the control group. The results of the hyperoxic group in the present study — significantly reduced alveolar number and surface area but no increase in Lm or mean alveolar volume — are probably the result of a smaller degree of lung inflation during fixation. Less pronounced inflation leaves the alveolar number unaffected but leads to reduced surface area and airway volume estimations. The most probable reason for this was hyperoxia-induced inflammation and edema formation. Both factors are known to inactivate pulmonary surfactant and, thereby, reduce lung distensibility [49, 50]. This is well reflected by the higher intra-alveolar volumes of inflammatory cells and edematous fluid in combination with reduced static compliance and elevated resistance and elastance. Hyperoxia-induced lung injury in adult baboons led to similar morphological alterations and was relieved by exogenous surfactant treatment [51].

Besides the parenchymal changes, the present study also provides evidence for an involvement of larger sized arteries and veins in the hyperoxia model of BPD. Arteries and veins larger than 25 *μ*m in diameter were grouped into a single compartment because previous studies using 3D reconstructions of the vasculature have shown that it may be impossible to distinguish morphologically between small arteries and veins in single 2D sections [52, 53]. Nevertheless, the data clearly show that the lumen of the blood vessels was narrower and the thickness of the wall as well as the perivascular space were increased in hyperoxic rabbits. These data complement the observations by Jiménez et al. [25] who reported a smaller peripheral vessel density and a thicker media layer of peripheral arteries. Our current data indicate that besides the peripheral arteries, remodeling of more centrally located blood vessels also takes place in the hyperoxia model and may additionally contribute to the pulmonary hypertension observed in this model [25, 54].

## 5. Conclusions

The present study provides robust stereological reference data for the preterm rabbit hyperoxia model of BPD. The data confirm and extend previous morphometric studies in this animal model and fit well with the pathological features of human BPD. In contrast to their normoxic counterparts, the lungs of preterm rabbits exposed to hyperoxia were characterized by reduced alveolarization, septal edema and/or fibroproliferation, thickened vessel walls, functional impairment of the parenchyma, and pulmonary hypertension. Together, these findings may serve as a reference to compare this model with BPD models in other species or future therapeutic interventions.

## Figures and Tables

**Table 1:** Summary of all acquired stereological parameters for both experimental groups. The data are presented as mean/STD.

Parameter	Normoxia group	Hyperoxia group
*V* (lung) [cm^3^]	3.773/0.437	2.314/0.398^∗^
*V*_V_ (par/lung)	0.898/0.028	0.809/0.046^∗^
*V* (par, lung) [cm^3^]	3.392/0.458	1.880/0.380^∗^
*V*_V_ (nonpar/lung)	0.102/0.028	0.191/0.046^∗^
*V* (nonpar, lung) [cm^3^]	0.381/0.092	0.434/0.090
*V*_V_ (airtot/par)	0.858/0.034	0.757/0.034^∗^
*V* (airtot, par) [cm^3^]	2.910/0.408	1.428/0.318^∗^
*V*_V_ (airduct/par)	0.274/0.024	0.285/0.042
*V* (airduct, par) [cm^3^]	0.931/0.166	0.528/0.095^∗^
*V* (airalv, par) [cm^3^]	1.979/0.265	0.901/0.275^∗^
*V*_V_ (sept/par)	0.142/0.034	0.243/0.034^∗^
*V* (sept, par) [cm^3^]	0.482/0.120	0.452/0.092
*V*_V_ (edema/airtot)	0.027/0.013	0.151/0.050^∗^
*V* (edema, airtot) [cm^3^]	0.075/0.032	0.208/0.053^∗^
*S*_V_ (sept/par) [cm^−1^]	558.4/70.55	475.7/49.73^∗^
*S* (sept, par) [cm^2^]	1896/367	893/201^∗^
$\bar{\tau }$ (sept) [*μ*m]	5.049/0.730	10.26/1.536^∗^
*N*_V_ (alv/par) [cm^−3^]	9.90 × 10^6^/0.98 × 10^6^	11.22 × 10^6^/2.08 × 10^6^
*N* (alv, par)	33.45 × 10^6^/4.70 × 10^6^	20.72 × 10^6^/3.98 × 10^6∗^
${\bar{\text{v}}}_{\text{N}}$ (alv) [*μ*m^3^]	60251/11761	45626/20195
Lm [*μ*m]	62.58/10.50	64.41/8.34
*V*_V_ (lumen/nonpar)	0.17/0.05	0.11/0.03^∗^
*V* (lumen, nonpar) [cm^3^]	0.061/0.014	0.048/0.011^∗^
*S*_V_ (vasc/lumen) [cm^−1^]	537/88	689/133^∗^
*S* (vasc, lumen) [cm^2^]	32.2/5.1	32.7/10.6
$\bar{d}$ (vasc) [*μ*m]	76.3/12.7	60.1/12.1^∗^
*V*_V_ (perivasc/nonpar)	0.086/0.022	0.143/0.029^∗^
*V* (perivasc, nonpar) [cm^3^]	0.032/0.010	0.063/0.019^∗^
*V*_V_ (vasc/nonpar)	0.042/0.011	0.044/0.009
*V* (vasc, nonpar) [cm^3^]	0.015/0.003	0.019/0.005
$\bar{\tau }$ (vasc) [*μ*m]	4.80/0.71	6.03/1.27^∗^
$\bar{\tau }$ (perivasc) [*μ*m]	9.93/2.27	19.53/4.33^∗^

^∗^Statistically different (*p* ≤ 0.05) from the normoxia group.

## Data Availability

Data are available on request.
